# Floating Thoracic Spine Due to Noncontiguous Fracture-Dislocations of the Thoracolumbar Spine

**DOI:** 10.7759/cureus.22955

**Published:** 2022-03-08

**Authors:** R Dinesh Iyer, Bhaskar Sarkar, Md. Quamar Azam, Pankaj Kandwal

**Affiliations:** 1 Orthopaedics (Trauma and Emergency), All India Institute of Medical Sciences Raipur, Raipur, IND; 2 Orthopaedic Surgery, All India Institute of Medical Sciences Rishikesh, Rishikesh, IND; 3 Trauma Surgery, All India Institute of Medical Sciences Rishikesh, Rishikesh, IND; 4 Orthopaedics, All India Institute of Medical Sciences Rishikesh, Rishikesh, IND

**Keywords:** spine trauma, floating spine, thoraco-lumbar spine, paraplegia, non-contiguous fracture-dislocations

## Abstract

The thoracolumbar spine is the most commonly afflicted area in vertebral column injuries. Here we bring up a case of a 20-year-old male who presented to our emergency department with a history of a high-velocity road traffic accident with noncontiguous two-level fracture-dislocations of the thoracolumbar spine with blunt trauma to the chest. The patient was managed with posterior reduction and instrumented postero-lateral fusion. Such an injury pattern has been reported only rarely in the literature. This report expects to highlight the unusual fracture pattern of a common injury and the challenges of managing such severe injuries intra-operatively and in the post-operative period.

## Introduction

The thoracolumbar region is the most commonly involved segment of the spine in traumatic spinal injuries [1]. The spectrum of the presentation can extend from anterior wedge compression (AO type A) without any neurological deficit to fracture-dislocations (AO Type C) with complete spinal cord injury (AIS grade A) [2]. The majority of these injuries involve T10 to L2 vertebrae, L1 being the most common amongst them.

We present a case of a young male who sustained noncontiguous fracture-dislocations of the thoracolumbar spine following an unrestrained high-velocity motor vehicle collision. Such an injury pattern has been reported quite infrequently; two case reports were involving the thoracic spine only and rest with lumbosacral and cervical involvement [3-8]. Written and informed consent was taken from the patient and his family members for using his individual and clinical data for publication and research purposes.

## Case presentation

A 20-year-old male was referred to our emergency department from a private hospital with a history of motor vehicle collision (unrestrained passenger in a four-wheeler that lost balance) that he sustained about 12 hours earlier. On primary survey as per ATLS protocol, the patient had decreased air entry on the right side of the chest with 94% oxygen saturation on room air. Ultrasound examination of the chest showed free fluid in the right thoracic cavity for which intercostal drainage was inserted with an underwater seal. Approximately 300mL blood was drained in the next six hours and the patient had maintained oxygen saturation since then on room-air. The blood pressure was recorded as 100/60 mm of Hg with a heart rate of 90 per minute (Grade 1 hypovolemic shock). After the primary survey, a thorough head-to-toe examination was done, focussing on any missed abdominal/pelvic organ injury or injuries to great vessels given the mode of injury and its severity. A contrast-enhanced CT scan was ordered to confirm the same.

The patient was unable to move bilateral lower limbs and there was a loss of sensation below nipple level. He had an in-situ urinary catheter inserted at the index hospital where he was first received. On clamping the catheter, there was no urge or discomfort and the clamp was removed timely.

A secondary survey revealed bruises over the chest wall on the right side and over the back on log rolling. The marked kyphotic deformity was seen in the thoracolumbar region and there were visible and palpable steps along spinous processes.

On neurological examination: motor, sensory and reflex components of upper limb were unremarkable; power was 0/5 (Medical Research Council grading) in all groups of muscles of lower limb; there was complete sensory loss (pinprick, touch, vibration, and joint position senses) below T5 dermatome, including lowest sacral segments; voluntary anal contraction and anal tone were absent; there was absent deep tendon reflexes in bilateral lower limbs; the plantar response was mute; bulbo-cavernous reflex was absent and could be attributed to spinal shock or possible injury to conus medullaris.

**Figure 1:**
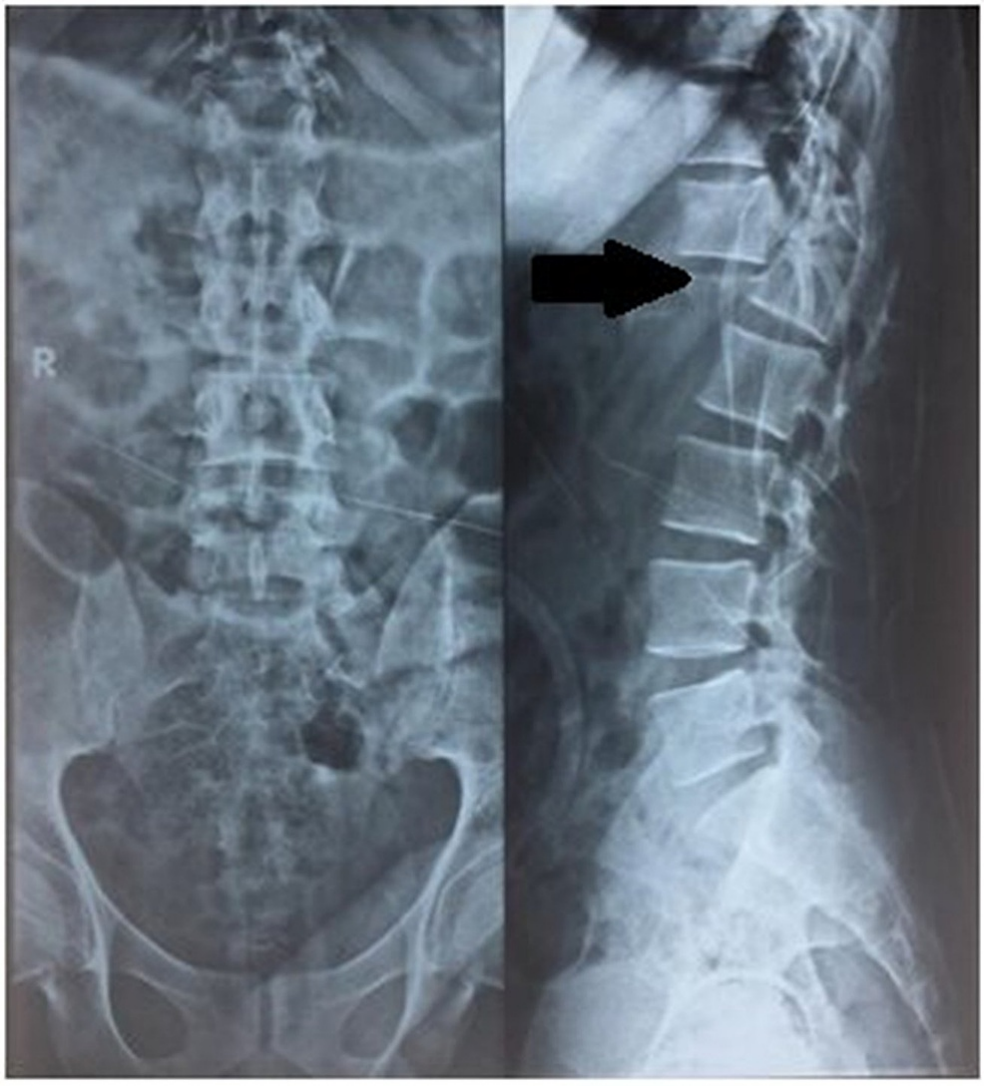
Preoperative radiograph (AP and lateral) of thoracolumbar spine showing T12-L1 fracture-dislocation (black arrow) AP - anteroposterior

**Figure 2:**
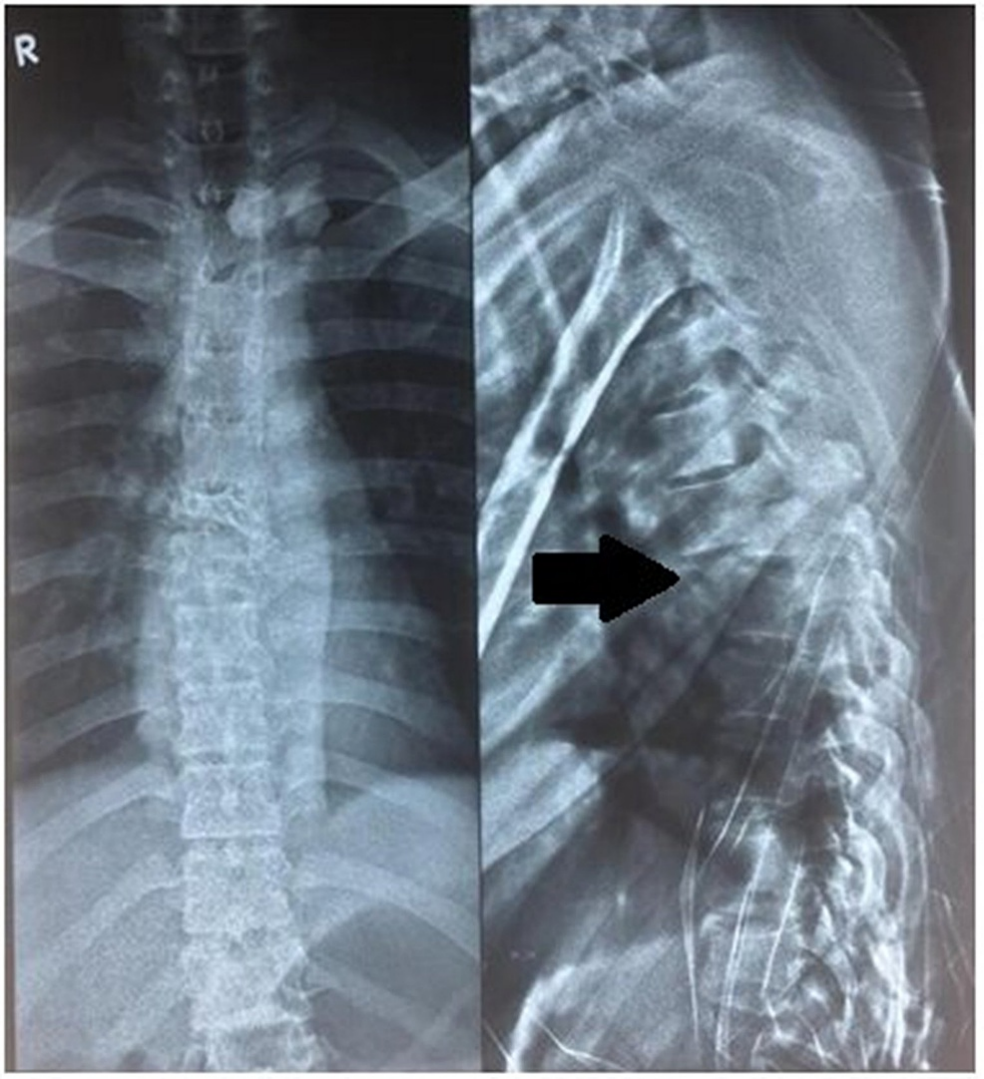
Preoperative radiograph (AP and lateral) of thoracic spine showing T6-T7 fracture-dislocation (black arrow) AP - anteroposterior

**Figure 3:**
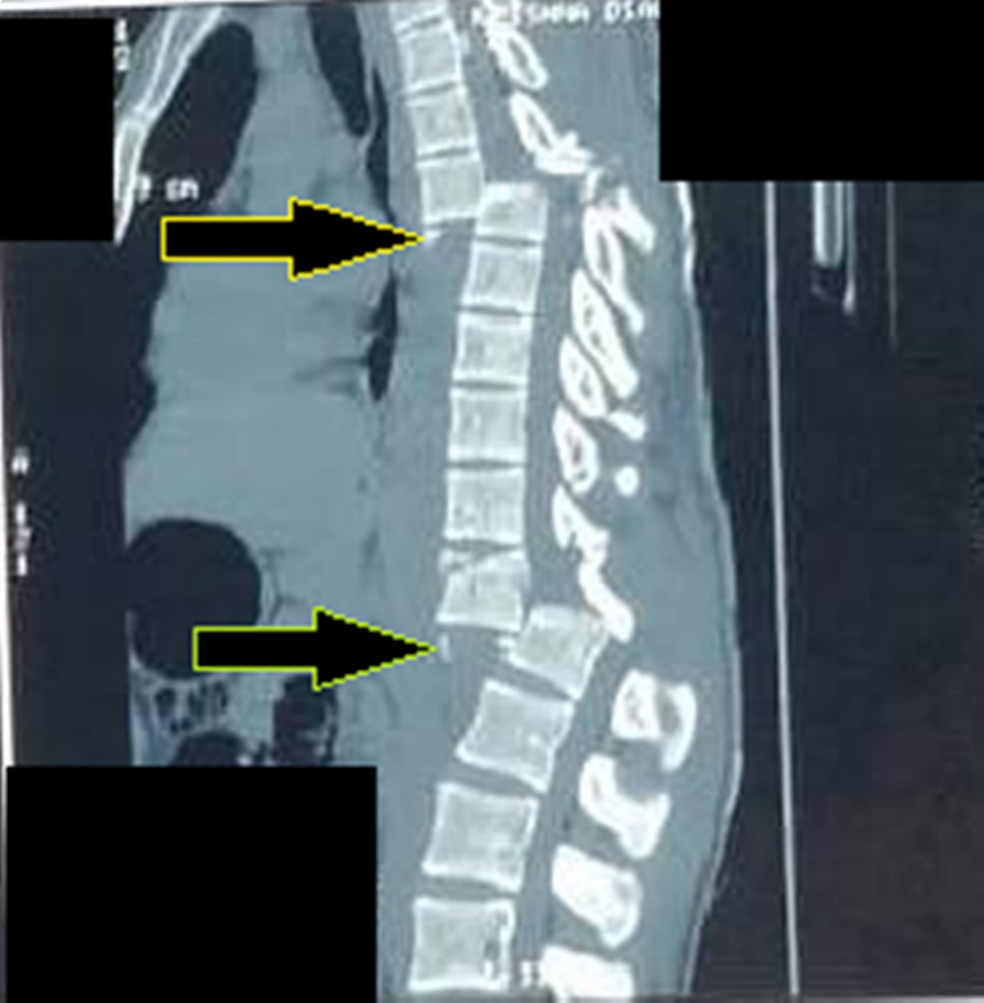
Preoperative mid-sagittal section of CT demonstrates the extent of translation at T6-T7 (proximal arrow) and T12-L1 (distal arrow)

**Figure 4:**
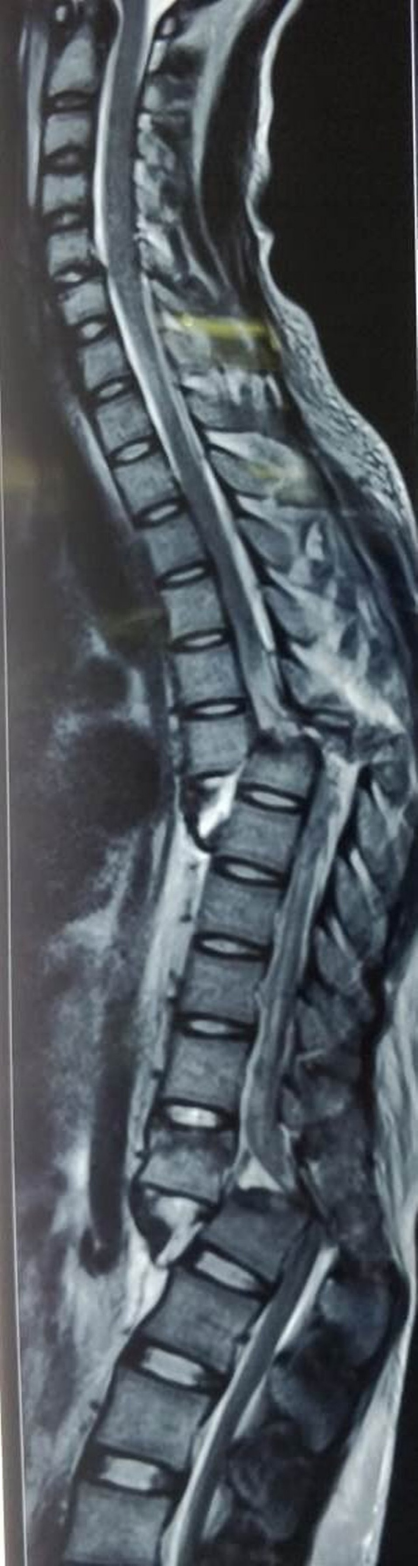
Preoperative sagittal T2-weighted MRI of spine showing the extent of damage to the spinal cord and posterior ligamentous complex

After adequate pain relief patient was sent for imaging studies with spinal protection. Orthogonal radiographs (Figures 1, 2) show the fracture-dislocations at two levels of the thoracolumbar spine: T6-T7 and T12-L1. The finding was confirmed by a CT scan. Figure 3 demonstrates the extent of the osseous injury on a sagittal section of the computed tomogram. Though MRI does not have much role in such trauma cases, it was done at the index hospital where he was first attended. Nevertheless, an idea of the magnitude of injury to the spinal cord can be obtained from the mid-sagittal section of the MRI (Figure 4).

On the basis of the mechanism of injury, clinical examination, and radiology, a diagnosis of “noncontiguous fracture-dislocations of the thoracolumbar spine (AO type C) involving T6-T7 vertebrae and T12-L1 vertebrae with spinal shock associated with right-sided hemothorax and multiple rib fractures” was made.

**Figure 5:**
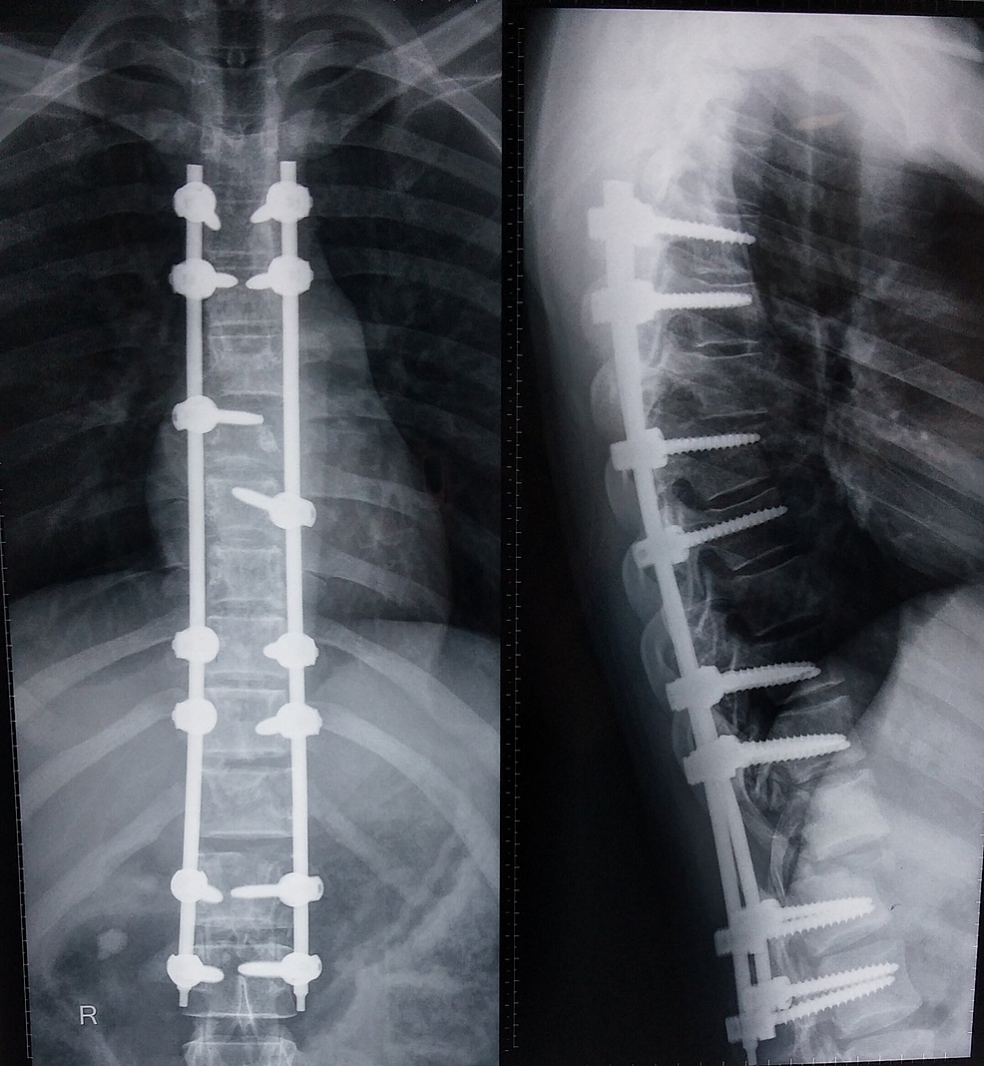
Postoperative radiograph following reduction and long-segment fixation

The patient and his family were informed about the dismal neurological prognosis and explained the need for surgery to facilitate rehabilitation. Written, informed consent was obtained and the patient was taken to the operating room on the same day. Under general anesthesia and in a prone position, posterior reduction and fixation with pedicle screws and rods were done and autograft from the left posterior-superior iliac crest was used for segmental postero-lateral fusion [9-11]. Figure 5 shows immediate postoperative radiographs.

Conscientious efforts were made for pain relief and vigorous chest physiotherapy from the immediate postoperative period. The patient was mobilized in a wheelchair with customized thoracolumbosacral orthosis from the first postoperative day. A chest drain was removed on the fourth postoperative day after minimal drainage for two consecutive days.

The patient was discharged on the fifth postoperative day with all instructions for care of paraplegia and chest injury as per protocol. At a two-week follow-up, the bulbo-cavernous reflex was present (favoring a diagnosis of complete cord injury and dissolution of spinal shock). At the three-month follow-up, the patient was able to transfer himself to a wheelchair independently and ambulate. He had been using clean intermittent self-catheterization for voiding and there was no pressure sore with a healthy surgical site. He had no complaints related to chest trauma and spinal implants at the final follow-up of 12 months.

## Discussion

AO type C injuries are usually associated with neurologic deficits, which are typically complete spinal cord injuries [10]. Therefore, patient and their family should always be informed about the guarded neurological prognosis and explained in detail about the high incidence of chronic complications like pressure sores, pulmonary infections, joint contractures, poor bladder-bowel habits in these patients. A multi-disciplinary approach from the initial presentation to treat and rehabilitate patients with such injuries can improve the outcomes significantly. Surgery for stabilization of the spinal column in these patients improves outcomes by aiding early mobilization, decreasing long-term complications, and improving psychological well-being with proper rehabilitation [12,13].

In our case, intra-operatively significant bleeding was encountered during exposure. There was extensive internal degloving, and soft-tissue handling was as meticulous as possible. Reduction screws were used to achieve a reduction of the proximal dislocated level (T6-T7) and the floating segment was first secured to the proximal intact cervicothoracic segment with a unilateral rod.

Then the distal fracture-dislocation was addressed and the body of T12 could not be completely reduced as it was locked over an anterior body of L1. PLC and posterior elements were already shattered and ends of transacted cord could be visualized from the defect. The dural tear was repaired using prolene 5-0 suture and augmented with fibrin glue. Efforts to achieve anatomical reduction were limited due to the poor general condition of the patient under anesthesia as well as excessive bleeding from surrounding degloved soft tissues and consequent physiological compromise. Moreover, the risk of major vessel injury with excessive blind manipulation and the rehabilitative nature of the surgical procedure made us prefer early closure over anatomic reduction which may have put the patient's life in danger. To prevent implant failure, we used autograft from the posterosuperior iliac crest for posterolateral fusion. 

Noncontiguous fractures of vertebrae are quite common with incidence varying from 3% to 20% in the literature. However, almost all such noncontiguous injuries have compression or burst fracture or may have one dislocated segment. An injury pattern with fracture-dislocation at two noncontiguous sites is very rare. This type of injury, akin to “floating spine,” involving a segment of the spinal column has been reported twice, though without associated other-system injuries [3,4]. Table 1 summarizes the existing literature on such multi-level spinal injuries.

**Table 1:** Overview of literature on noncontiguous fracture-dislocations of the spine

Serial no.	Authors	Journal	Mode of injury	Level of injury and neurological status	Management	Neurological outcome
1.	Salehani, et al. [3]	World Neurosurgery, 2016	Fall from height (30 feet)	T5-T6 T9-T10 Three column injury with fracture-dislocations at both levels (AO type C) AIS grade A neurology	Posterior only approach followed by additional anterior instrumentation and fusion after 6 months due to implant failure.	AIS grade A at follow-up.
2.	Deokate, et al. [4]	International Journal of Scientific Study, 2017	Fall from stairs under influence of alcohol	T6 - Anterior wedge compression with intact posterior cortex (AO type A1) T12 - Burst fracture with retropulsed fragment ( AO type A4) Patient in spinal shock at presentation	Posterior decompression, T12 corpectomy and long segment fixation using posterior only approach	AIS grade C at follow up
3.	Cho SK, Lenke LG, Hanson D [5]	The Spine Journal, 2006	Motor vehicle accident	L2-L3 L5-S1 Fracture dislocations at both levels (AO type C) Incomplete spinal cord injury corresponding to cauda equina syndrome	Two stage procedure within a week - 1. Open reduction of the L2–L3 and L5–S1 fracture-dislocations, posterior spinal fusion with instrumentation, and sacropelvic fixation 2. Anterior spinal arthrodesis of L5–S1 using titanium mesh cage and autologous iliac crest bone graft	Ambulatory patient with improvement of ankle dorsiflexion (4/5) bilaterally ( No comments on bulbocavernous reflex or anal tone at follow up)
4.	Csókay, et al. [7]	Spinal Cord, 2001	Motor vehicle accident	C7 - Burst fracture T4-T5 - Fracture dislocation C7-T3 - Incomplete spinal cord injury (AIS grade B, partail sensory preservation) Below T4 - Complete injury (AIS grade A)	C7- Anterior cervical corpectomy, fusion and fixation using anterior cervical plate (Titanium Orion plate and screws; Sofamor-Danek) done within 6 hours of injury T4 - Anterior decompression by T4 body resection and disc removal, cortico-cancellous bone grafting and T3 ± 5 ventral titanium Z plate fixation (Sofamor- Danek) were done via a right upper transthoracic approach within 10 h	At six months follow up he could walk with one stick and one year later he returned to his profession with slight paraparesis. His sphincter and sexual functions also returned completely after 1 year (AIS grade D)

Although rarely encountered, through this case we intend to emphasize the importance of properly addressing frequently associated systemic injuries with this kind of spinal trauma, diligent soft-tissue handling during surgery, consideration to “second-hit” from prolonged surgery notwithstanding nonanatomic reduction, and multi-disciplinary rehabilitation while treating these patients.

## Conclusions

Noncontiguous fracture-dislocations of the thoracolumbar spine akin to a “floating thoracic spine” are rare injuries with poor outcomes. Primary management of trauma patients using a systematic approach like ATLS helps in addressing life-threatening injuries and reduces incidences of missed injuries.

In this case, the reduction and long-segment fixation of the thoracolumbar spine has given good clinical results. Surgery for stabilizing the spinal column in these patients is not meant primarily for neurological outcomes but to improve their quality of life by aiding early mobilization, decreasing long-term complications, and improving psychological outcomes with proper rehabilitation. Early surgical stabilization with a multidisciplinary approach involving urologists, physical therapists, and psychologists is the way forward in the management of patients with such devastating injuries.
